# Zinc Chelation Mediates the Lysosomal Disruption without Intracellular ROS Generation

**DOI:** 10.1155/2016/6724585

**Published:** 2016-03-30

**Authors:** Andreza Cândido Matias, Tânia Maria Manieri, Giselle Cerchiaro

**Affiliations:** Center for Natural Sciences and Humanities, Federal University of ABC, UFABC, Avenida dos Estados 5001, Bloco B, 09210-170 Santo André, SP, Brazil

## Abstract

We report the molecular mechanism for zinc depletion caused by TPEN (N,N,N′,N′-Tetrakis(2-pyridylmethyl)ethylenediamine) in neuroblastoma cells. The activation of p38 MAP kinase and subsequently caspase 3 is not due to or followed by redox imbalance or ROS generation, though these are commonly observed in literature. We found that TPEN is not responsible for ROS generation and the mechanism involves essentially lysosomal disruption caused by intracellular zinc depletion. We also observed a modest activation of Bax and no changes in the Bcl-2 proteins. As a result, we suggest that TPEN causes intracellular zinc depletion which can influence the breakdown of lysosomes and cell death without ROS generation.

## 1. Introduction

N,N,N′,N′-Tetrakis(2-pyridylmethyl)ethylenediamine (TPEN) is a membrane-permeable hexadentate compound which chelates metal ions such as Cu^2+^, Zn^2+^, and Fe^2+^ [1, 2], but this intracellular chelator has shown a high affinity for zinc [3–5]. This special property permits the use of TPEN in a variety of settings, often as a tool to probe the functions of zinc in cell cultures.

Studies using TPEN have demonstrated that intracellular zinc depletion causes oxidative stress and DNA damage [6], as well as apoptosis in some cells in culture [5, 7, 8]. TPEN could also inhibit the neurotoxic effects of zinc* in vivo* [9, 10]. Previous studies have shown that TPEN induced ROS formation by intracellular zinc depletion and, consequently, DNA damage [4, 6] and apoptosis [7–11]. The most known mechanism involves the activation of caspase 11 [12], caspase 3/7 [4], p53 [13], cleaved PARP, and apoptotic bodies [4]. It has been proposed that zinc deficiency can cause increased oxidative stress and, consequently, cell damage or death [14].

The effects of zinc depletion in cells have been widely discussed in literature, and TPEN is one of the most functional chelators used in those studies. The activation of p38 by an excess of zinc has demonstrated that this mitogen-activated protein kinase (MAP kinase) is responsible for the zinc-mediated activation of the mRNA expression of the Th1 cytokines interferon-gamma and interleukin-2 in human T-cells [15, 16]. Zinc was proven to be involved in apoptosis via the activation of a p38 MAP kinase pathway triggered by reactive oxygen species (ROS) and redox regulation [17–19]. The ubiquitination induced by excess zinc also required p38 activation in neuronal cells [20], and this kinase is activated by treatment with a Zn ionophore complex in HL-60 cells [19]. Conversely, the commitment of intracellular zinc deficiency to the regulation of cell death is an intriguing matter to study. When chelation therapy limits zinc access in cultured cells, DNA synthesis ceases, the cell cycle is arrested [21], a redox imbalance can be established [22, 23], and the involvement of both p53 and caspase 11 has been proposed [13, 24]. However, the molecular mechanism of cell death caused by zinc deficiency is not fully understood. Therefore, the use of intracellular specific chelators, such as TPEN, is also very helpful to understand zinc biology.

For that reason, in the present study, we have investigated the detailed mechanisms of the cell death pathway caused by the specific zinc chelator TPEN.

## 2. Materials and Methods

### 2.1. Cell Culture

Human neuroblastoma SH-SY5Y cells were purchased from the American Type Culture Collection (ATCC) and grown in Dulbecco's Modified Eagle F12 Medium (DMEM/F12) supplemented with 10% heat-inactivated fetal bovine serum (Gibco) and antibiotics as described [25]. The cells were routinely trypsinized and seeded at a density of 4 × 10^4^ cells/cm^2^. Every month, the cells were cultivated in the absence of antibiotics for control purposes and subjected to a routine assay using a MycoAlert Mycoplasma Detection Kit (Lonza Rockland) to ensure that they had not become contaminated with mycoplasma. All SH-SY5Y cells in this study were used at a low passage number (<20).

### 2.2. Cell Viability Assessment

To determine the levels of TPEN that would promote cell death, concentration-dependent cytotoxicity studies were performed. Typically, the viability of neuroblastoma cells was assessed by 3-(4,5-dimethylthiazol-2-yl)-2,5-diphenyltetrazolium bromide (MTT) reduction assays, as previously reported [26]. SH-SY5Y cells were inoculated in 96-well plates at a density of 8 × 10^4^ cell/cm^2^ and incubated for 24 hours under the conditions described above. Aliquots of freshly prepared solutions of TPEN (2.5 mM) were added to the culture medium to attain final concentrations in the 5.0–100.0 *μ*M range, and the plates were then incubated for an additional 12, 24, and 48 hours. The plates were also preincubated with 10 *μ*M SB202190 (p38 MAPK inhibitor) and/or 300 *μ*M antipain dihydrochloride (A.D.: cathepsin inhibitor) for 1 hour and subsequently treated with 25 *μ*M TPEN for 12, 24, and 48 hours. Trypan Blue dye exclusion test was performed to confirm the MTT assay results. SH-SY5Y cells were inoculated in 24-well plate at a density of 4 × 10^4^ cell/cm^2^ and incubated for 24 hours under the conditions described above. Aliquots of freshly prepared solutions of TPEN (2.5 mM) were added to the culture medium to attain final concentrations in the 5.0–100.0 *μ*M range, and the plates were then incubated for an additional 48 h. Following incubation, the cells were trypsinized and combined, washed with phosphate buffered saline (PBS; 137.0 mM NaCl and 2.7 mM KCl in 10 mM phosphate buffer at pH 7.4), stained with Trypan Blue, and counted under an optical microscope using a Neubauer chamber.

### 2.3. Determination of Cellular Zinc by Flame Atomic Absorption Spectroscopy (FAAS)

The determination of intracellular zinc concentrations was made as previously reported [25]. We employed a flame atomic absorption spectrometer, Model AAS Vario 6 (Analytik Jena AG, Jena, Germany), equipped with a hollow zinc cathode lamp and a deuterium lamp for background correction. SH-SY5Y cells were plated in a 25 cm^2^ culture flask at a density of 8 × 10^4^ cells/cm^2^ and incubated in the presence or absence of TPEN (5 or 25 *μ*M) for 6, 24, and 48 hours. After incubation, the cells were trypsinized and combined, washed twice with PBS containing 1.0 mM EDTA to remove residual Zn(II), washed three additional times with PBS, dried for 1 week in a desiccator, and then analyzed by FAAS.

### 2.4. Cell Death Assay

The percentage of cells undergoing apoptosis and necrosis was determined by Annexin V staining using the ApopNexin*™* FITC Apoptosis Detection Kit (Millipore) in a flow cytometer (Cytometer FC 500 MPL, Beckman Coulter). SH-SY5Y cells were seeded in 6-well plates and treated for 12, 24, and 48 hours with 5 or 25 *μ*M TPEN and 100 nM staurosporine (positive control). The apoptosis assay was performed according to what is described in Matias et al. [25]. Apoptosis assays were performed at least 7 times in independent replicate experiments.

### 2.5. Measurement of Intracellular Reactive Oxygen Species

SH-SY5Y cells that had been plated and incubated in the presence or absence of TPEN (5 or 25 *μ*M) as described above were treated with a trypsin/EDTA (1 mM) solution, washed three times with PBS, and resuspended in a 50.0 *μ*M solution of the oxidation-sensitive, nonfluorescent probe 50 *μ*M 2′,7′-dichlorodihydrofluorescein diacetate (DCFH-DA) [27] with PI [27, 28] or 5 *μ*M dihydroethidium (DHE). 300 *μ*M H_2_O_2_ and 50 *μ*M DMNQ were used for positive control to DCFH-DA and DHE, respectively. The cells were washed three times with PBS after incubation at 37°C for 45 min [29] to probe DCFH-DA and at 37°C for 30 min to probe DHE, and the levels of intracellular fluorescence were determined immediately by flow cytometry at 530 nm using a Cytometer FC 500 MPL (Beckman Coulter) [30, 31]. Assays were conducted at least in quintuplicate, and >20,000 viable cells from each sample were analyzed per assay.

### 2.6. Western Blot Analyses

SH-SY5Y cells were plated and incubated in the presence or absence of TPEN (5 or 25 *μ*M) as described above. Then, at 6, 12, and 24 hours after treatment, the cells were harvested, resuspended and lysed in 150 *μ*L of RIPA buffer (150 mM NaCl, 5 mM EDTA, 1 mM dithiothreitol, 1% Triton X-100, 0.5% sodium deoxycholate, and 0.1% SDS in 50 mM Tris at pH 7.5) containing a protease inhibitor cocktail for mammalian cells (Sigma-Aldrich), and centrifuged (14000 g, 20 min, 4°C). The supernatants and pellets were transferred to new microcentrifuge tubes and stored at 80°C until required for analysis. The protein concentrations were determined following Lowry's method using bovine serum albumin (BSA) as the standard [32]. Then, 100 *μ*g of extracts was subjected to SDS-PAGE and blotted onto nitrocellulose membranes (GE Healthcare Life Sciences) with the equal loading of the proteins confirmed by the internal mass control blotting of *β*-actin or *α*-tubulin. The membranes were blocked for 1 hour in a blocking solution containing 5% nonfat dried milk (Sigma-Aldrich) and 0.0025% sodium azide solubilized in TBS-T (150 mM NaCl, 50 mM Tris at pH 7.5 and 0.05% Tween-20) and then washed twice with TBS-T. The primary antibodies employed were the rabbit anti-caspase 3 (Sigma-Aldrich), mouse anti-*β*-actin (clone 279 AC-74; Sigma-Aldrich), mouse anti-p38 (A-12: sc-7972; Santa Cruz Biotechnology), rabbit anti-JNK/SAPK1 (Millipore), rabbit anti-Bax (NT, Millipore), mouse anti-Bcl-2 (clone 100, Millipore), mouse anti p-p38 (D-8: sc-7973, Santa Cruz), mouse anti-JNK/SAPK1 (pT183/pY185; clone 41/JNK/SAPK 14 pT183/pY185; BD Biosciences), and mouse anti-*α*-tubulin (DM1A: sc-32293). The protein complexes that were formed following treatment with the specific secondary antibodies (anti-mouse or anti-rabbit IgG-peroxidase conjugate) were detected using the chemiluminescent substrate (Thermo Fisher Scientific). Western blottings were conducted at least in triplicate and represent independent replicate experiments.

### 2.7. Acridine Orange Assay

SH-SY5Y cells were plated and incubated in the presence or absence of TPEN (5 or 25 *μ*M) as described above. At 4, 12, and 24 hours after treatment, the cells were incubated with Acridine Orange (2.5 *μ*g/mL) in DMEN/F12 medium for 15 min at 37°C. After incubation, the cells were trypsinized and washed three times with PBS, and the red (FL3, 670 nm) fluorescence was recorded on a logarithmic scale by flow cytofluorometry using a Beckman Coulter Quanta SC MPL instrument excited at 488 nm. Untreated but AO-stained cells, which were used as control, had a fluorescence intensity set between 10^2^ and 10^3^ a.u. on a logarithmic scale. As lysosomes are being degraded there is a decrease in the red fluorescence peak and a simultaneous increase of weaker red fluorescence (subpopulation) under 10^2^ a.u. Subpopulations of red fluorescence were evaluated to observe the lysosomal integrity/rupture.

### 2.8. Statistical Analysis

All experiments were repeated at least three times in independent replicates (except where stated otherwise), and the results are expressed as the mean values ± standard deviations. The analysis of variance (ANOVA) with Bonferroni's correction was used to evaluate the differences between the means, with the level of significance set at *p* < 0.05.

## 3. Results and Discussion

### 3.1. TPEN Causes a Decrease in Cell Viability and Intracellular Zinc Levels in Neuroblastoma Cells SH-SY5Y

**Figure 1:**
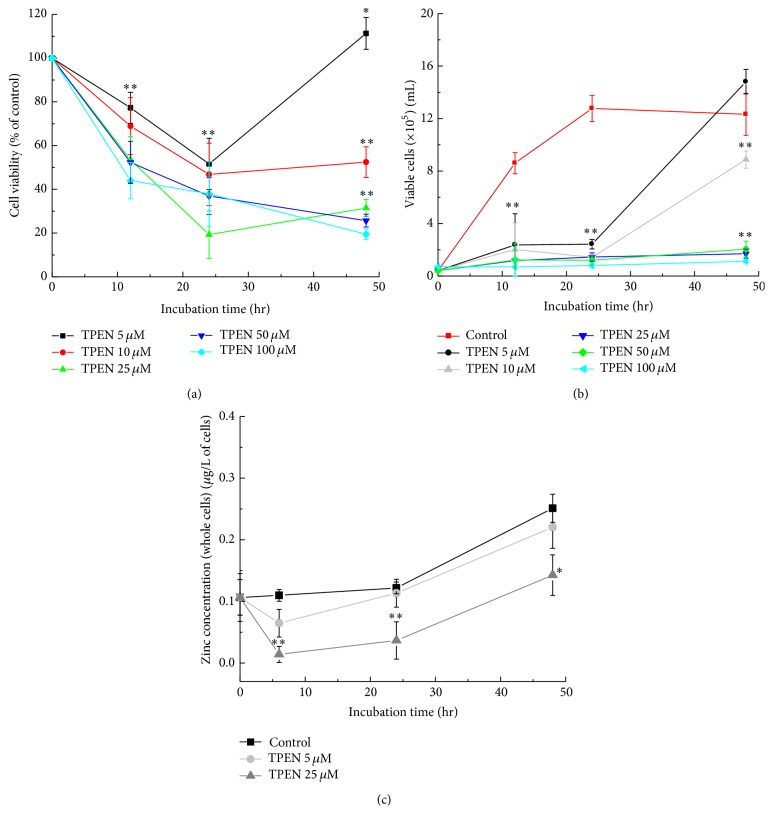
Influence of the TPEN chelator on cell viability and intracellular zinc alterations. (a) Concentration-dependent studies for MTT assay on neuroblastoma. SH-SY5Y cells were treated with varying concentrations of TPEN, while untreated cells were used as control. (b) Concentration-dependent studies for Trypan Blue assay on neuroblastoma. SH-SY5Y cells were treated with varying concentrations of TPEN, while untreated cells were used as control. (c) The concentration of zinc was determined by flame atomic absorption spectroscopy. Data represent the mean values ± standard deviation (*n* = 3), and significant differences between treated and untreated cells were ^*∗*^ *p* < 0.05 and ^*∗∗*^ *p* < 0.001, respectively.

Several studies have indicated that the metal chelator TPEN induces intracellular zinc depletion in several cell types [5, 33, 34] and here the effects of chelator TPEN on the SH-SY5Y neuroblastoma cell viability were assessed using the MTT assay. Raw data from Viability by MTT method are provided in the Supplementary Material available online at http://dx.doi.org/10.1155/2016/6724585. We selected concentrations of 5 and 25 *μ*M TPEN based on the opposite dose-dependent profiles that they can cause in cells (Figure 1(a)).

It was observed that cells treated with 5 *μ*M TPEN showed an increase in cell viability after 48 hours. On the contrary, cells treated with 25 *μ*M TPEN showed a decrease in cell viability during the 48 hours of treatment. The results of the Trypan Blue assay showed the same profile for cells treated with 5 *μ*M TPEN and 25 *μ*M TPEN (Figure 1(b)). The zinc concentration was precisely quantified by flame atomic absorption spectrometry (FAAS) [1]. The decreasing cell viability observed in cells treated with TPEN is related to the changes in the intracellular zinc levels. Our previous studies showed that TPEN can disrupt the intracellular zinc levels in mammalian cells but it cannot disrupt either copper or iron levels [3]. The results point that intracellular zinc levels in cells treated with 25 *μ*M TPEN decreased compared with the control cells (cells with no chelator, Figure 1(c)), whereas cells treated with 5 *μ*M TPEN showed no significant changes in the zinc levels. This concentration showed no toxic effect in cells, as predicted by many previous studies that used even higher concentrations to chelate zinc [35–41]. Our results showed that neuroblastoma cells treated with the intracellular zinc chelator TPEN had a decrease in cell viability. However, after 48 hours of incubation, it was observed that cells treated at a concentration of 5 *μ*M are able to “break” the barrier and return to viability, indicating that a reservoir of intracellular zinc may exist; conversely, cells treated with high TPEN concentrations (25, 50, and 100 *μ*M) showed a lower cell viability. Therefore, we chose the lowest working concentration to relate changes in intracellular zinc deficiency and the molecular consequences in neuroblastoma cells.

### 3.2. Both Necrosis and Apoptosis Are Caused by TPEN Inducing Intracellular Zinc Depletion

**Figure 2:**
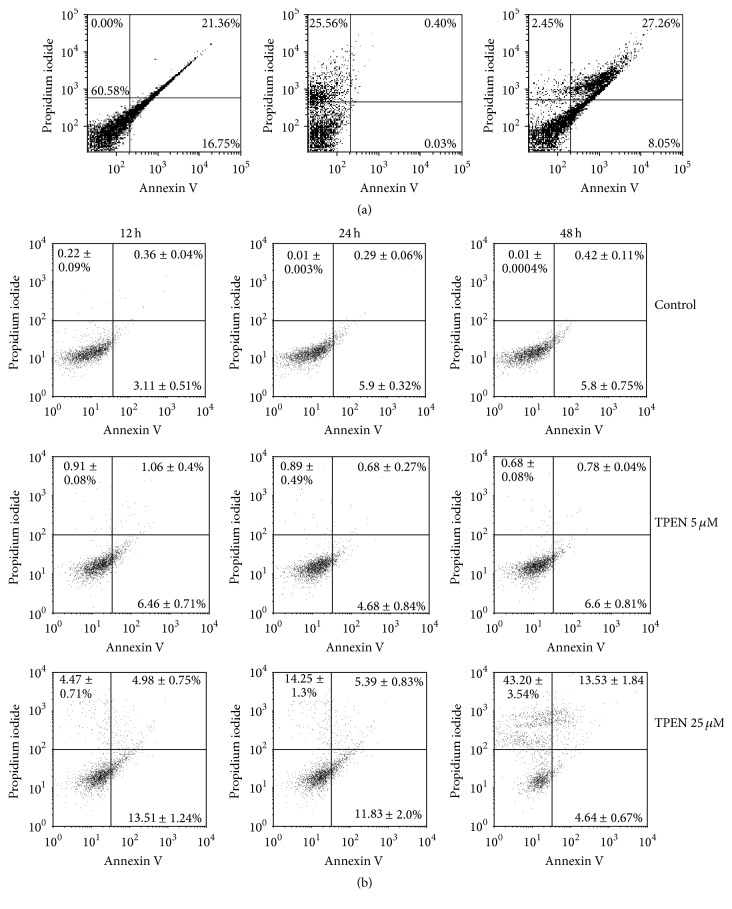
Apoptosis analysis. (a) Dot plot of SH-SY5Y after exposure to 100 nM staurosporine for 4 hours (positive control). Dot plot graphs from left to right show cells treated with (1) 100 nM staurosporine labeled with Annexin V-FITC, (2) 100 nM staurosporine labeled with PI, and (3) 100 nM staurosporine labeled with Annexin V-FITC and PI. (b) Dot plot of SH-SY5Y after exposure to 5 or 25 *μ*M TPEN for 12, 24, and 48 hours and flow cytometry analysis with Annexin V-FITC versus PI. The divisions of the plots distinguish necrotic cells (Annexin V^−^/PI^+^, left upper quadrant) from early apoptotic cells (Annexin V^+^/PI^−^, right lower quadrant) and late apoptotic cells (Annexin V^+^/PI^+^, right upper quadrant). The plots in the figure are representative of five independent experiments. Data represent the mean values ± standard deviations (*n* = 3) and significant differences between untreated cells and cells treated with TPEN 25 *μ*M.

Because cells treated with 25 *μ*M TPEN showed both decreasing cell viability and intracellular zinc depletion, we correlated these effects with the mechanism of cell death. The results were obtained by flow cytometry analysis with Annexin V labeling FITC (axis *x*) and propidium iodide (PI) (axis *y*) and were interpreted as necrotic cells (Annexin V^−^/PI^+^, left upper quadrant) from early apoptotic cells (Annexin V^+^/PI^−^, right lower quadrant) and late apoptotic cells (Annexin V^+^/PI^+^, right upper quadrant). Positive control was done by 100 nM staurosporine for 4 hours (Figure 2(a)). Cells treated with 25 *μ*M TPEN showed 13.5% of the cells in the early stages of apoptosis and 4.5% of the cells in necrosis after 12 hours of incubation (Figure 2(b)). However, after 48 hours of incubation, we observed that the percentage of necrotic cells increased to 43.2%, whereas the cells treated with 5 *μ*M TPEN showed no significant results compared with the control cells (Figure 2(b)). The results of flow cytometry revealed that, depending on the time of exposure, zinc depletion caused cell death by either necrosis or apoptosis, with the most part occurring via necrosis. Necrosis was observed as an accidental and unregulated cellular event. However, evidence suggests that both necrosis and apoptosis may also occur by regulation mechanisms that are normally initiated by TNF-*α*, Fas, or TRAIL and mediated the formation of the two kinase complexes RIP3 and RIP1 [42–45]. It is also known that other signal-controlled mitochondrial dysfunctions such as generation of ROS, ATP depletion, proteolysis by calpains and cathepsins, and membrane rupture processes are also involved in necrosis [46]. Subsequently, we quantified the levels of ROS and lysosomal integrity to understand the mechanism involved in cell death by necrosis when zinc is depleted by TPEN.

**Figure 3:**
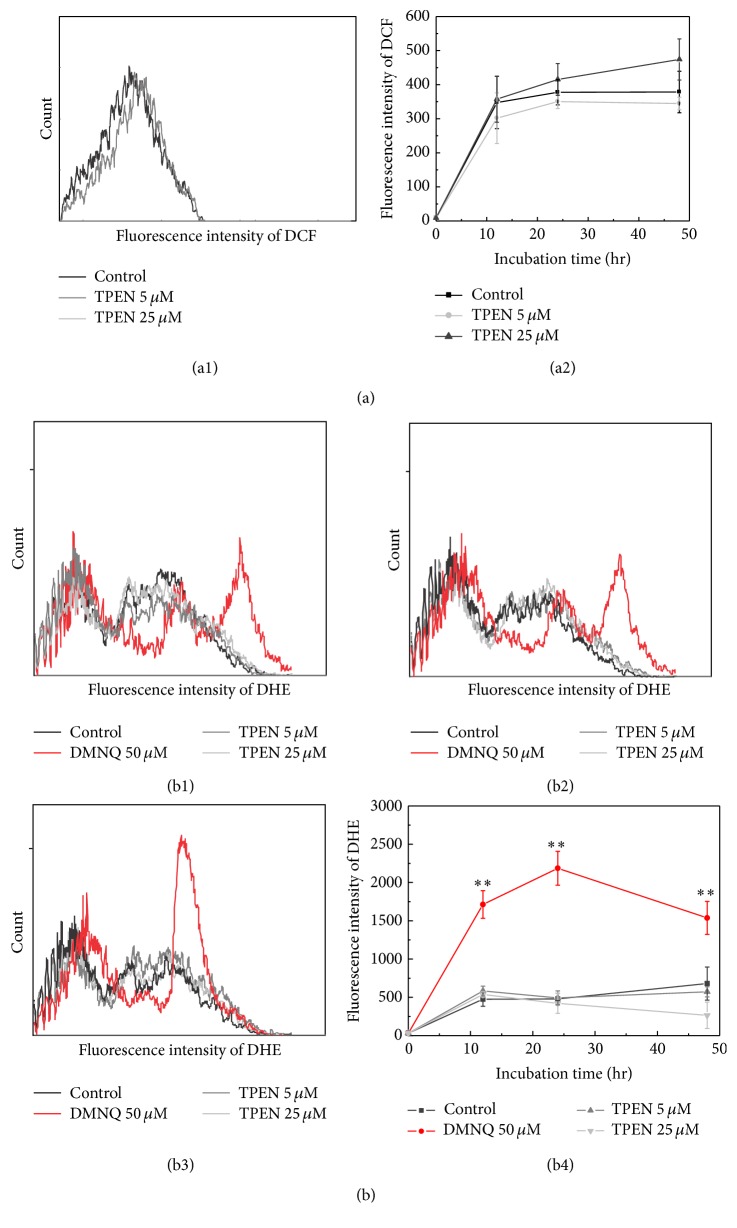
ROS Analysis. (a) Intracellular fluorescence of 2′,7′-dichlorodihydrofluorescein (DCF) in SH-SY5Y cells that were treated or not with 5 or 25 *μ*M TPEN for 12, 24, and 48 hours measured by FACS (a1) flow cytometric data compiled on a single graphic showing no change in DCF fluorescence in different incubation time and concentration of TPEN (a2). Viable cells were labeled with PI to exclude dead cells and only viable cells were analyzed. (b) Dihydroethidium (DHE) in SH-SY5Y cells treated with 5 or 25 *μ*M TPEN for 12 (b1), 24 (b2), and 48 hours (b3). Flow cytometric data compiled on a single graphic showing no change in DHE fluorescence in different treatment time and concentration of TPEN (b4). Data represent the mean values ± standard deviations (*n* = 3). Significant differences between positive control (DMNQ) and treated cell were ^*∗∗*^ *p* < 0.001. There were no significant differences between untreated and treated cells.

To analyze the influence of reactive oxygen species (ROS) on zinc depletion, cells were incubated with the probe DCFH-DA and PI and analyzed by flow cytometry [47]. Cells were treated with PI to discriminate dead cells from the viable ones. Viable cells were gated and the results indicated that both untreated cells (control) and those treated with TPEN exhibited the same fluorescence profile that remained unchanged throughout the incubation period of 12, 24, or 48 hours, indicating that there is no ROS generation to cause probe oxidation (Figures 3(a) and 3(b)). The results of the indirect quantification of ROS suggest that the activation of the observed cell death related to TPEN is not involved in redox imbalance, in contrast to many cell death processes.

### 3.3. Lysosomal Disruption Is Involved in Intracellular Zinc Depletion

**Figure 4:**
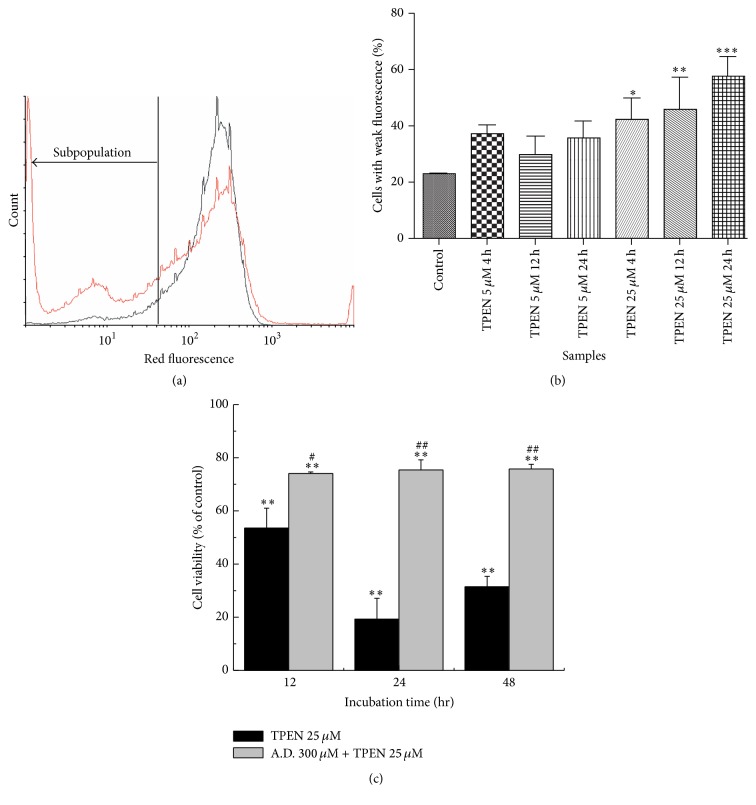
(a) Histogram of lysosomal disruption assay with Acridine Orange measured by flow cytometry. Black line shows control population and red line shows increase in the percentage of cells exhibiting a weaker fluorescence (subpopulation). (b) Subpopulation (weaker fluorescence) of 670 nm fluorescence analysis of SH-SY5Y after exposure to 5 or 25 *μ*M TPEN for 4, 12, and 24 hours. (c) SH-SY5Y cells were pretreated with 300 *μ*M antipain dihydrochloride (A.D.: cathepsins inhibitor) for 1 hour and subsequently incubated with 25 *μ*M TPEN for 12, 24, and 48 hours and analyzed by MTT assay. Untreated cells were used as control and treated cells with 25 *μ*M TPEN were used as positive control of cell death. Data represent the mean values ± standard deviation (*n* = 3), and significant differences between treated and untreated cells were ^*∗*^ *p* < 0.05, ^*∗∗*^ *p* < 0.001, and ^*∗∗∗*^ *p* < 0.0001; and significant differences between treated and 25 *μ*M TPEN treated cells were ^#^ *p* < 0.05 and ^##^ *p* < 0.001.

Since the decrease of intracellular zinc is not related to ROS but we do see cell death, one hypothesis is that cathepsins, proteins present in lysosome, can lead to cell death by necrosis once extravasated to the cytosol. We analyzed the lysosomal integrity by flow cytometry using the probe Acridine Orange (AO). AO is a metachromatic dye that shows different emission wavelengths when exposed to light at 488 nm. It interacts preferentially with acidic vesicles; for example, when the dye is found in high concentrations in the lysosome, these compartments are labeled dark red. When it occurs in a low concentration in the cytoplasm and nucleus, AO shows a green fluorescence. The lysosomal integrity is then monitored by the level of red fluorescence, so when the lysosomal integrity is disrupted, there is an increase in the percentage of cells with weak red fluorescence, also known as the subpopulation [47–51]. An increase in the percentage of cells exhibiting a weaker fluorescence (subpopulation) (Figure 4(a)) at 670 nm in cells treated with 25 *μ*M TPEN during 24 hours was observed, revealing that there was a high level of lysosomal rupture compared with control cells (Figure 4(b)). So it is possible to infer that intracellular zinc depletion is related to lysosomal rupture. AO intercalation into DNA and RNA is too low to disturb the lysosomal integrity assay [48]. There is still undisrupted lysosome, even in cells with moderate treatment. Therefore, the most effective measure of the rupture is in accordance with the change in fluorescence subpopulation, which is modified in the case of lysosomal lysis. The release of cathepsins is one of the consequences of lysosomal rupture, and this can trigger cell death by apoptosis. To demonstrate that the lysosomal rupture is an event intrinsically correlated to the chelating action of TPEN, it was used to a cathepsin inhibitor (antipain dihydrochloride (A.D.)). The MTT viability assay showed that cells pretreated with 300 *μ*M A.D. presented an increase in cell viability compared with treated cells with 25 *μ*M TPEN (Figure 4(c)). Therefore, it was concluded that TPEN-induced cell death is dependent on the cathepsin release.

### 3.4. TPEN Activated Bax, Caspase 3, and p38 MAPK

We also determined which pathways are involved in cell death. The JNK and p38 MAP families of protein kinases are involved in stress-induced apoptosis, and some reports have indicated that these kinases are also involved in necrotic cell death [52, 53]. It is also known that proteins of the Bcl-2 family are involved in apoptosis with mitochondrial dysfunction, in cell membrane rupture, and in necrosis, leading us to examine the involvement of Bcl-2 and Bax proteins in cell death. The p38 MAP kinase can also sensitize cells to apoptosis by overexpression of Bax [54, 55]. Therefore, the expressions of p38 MAP kinase and Bcl-2 were also analyzed to understand the mechanism of action of TPEN and its depletion of intracellular zinc.

**Figure 5:**
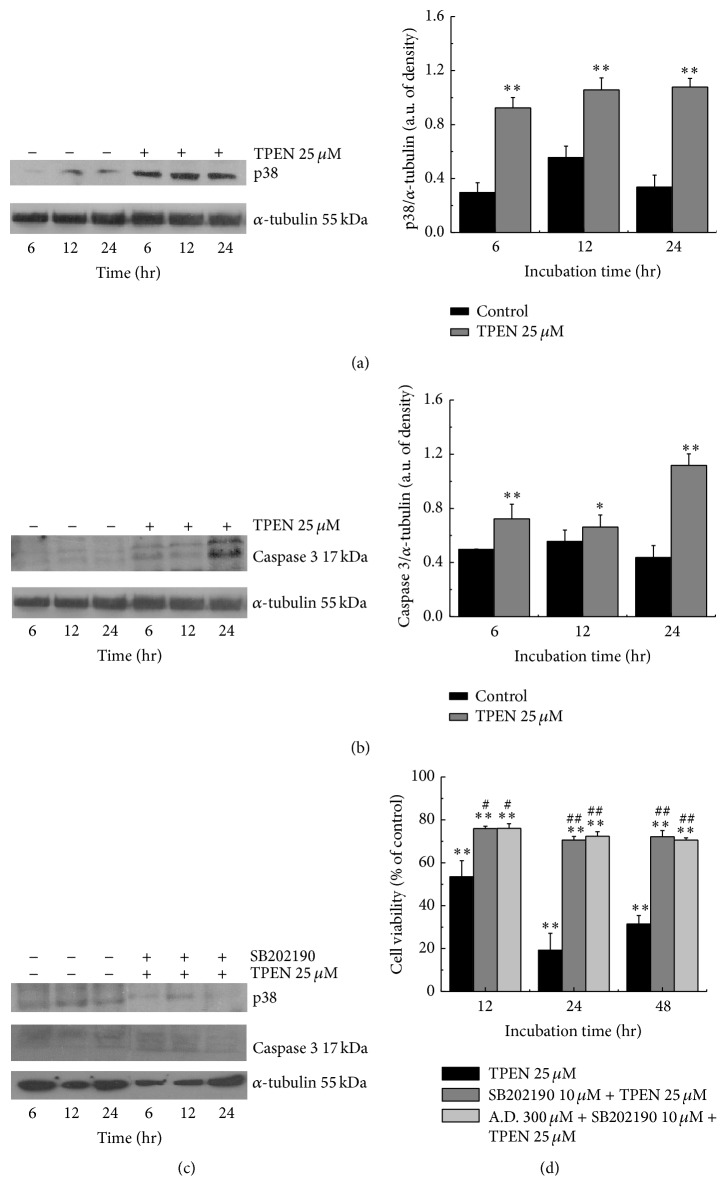
Western blot indicates whether expression levels of apoptotic proteins were upregulated or not during TPEN therapy with zinc depletion in neuroblastoma cells. SH-SY5Y cells were treated with 25 *μ*M TPEN. At each time point, 100 *μ*g of total proteins from the total cell lysates was loaded onto each lane for the detection of p38 (a and c) and caspase 3 (b and c). (c) The p38 MAPK and caspase 3 levels were determined after inhibition of the p38 protein with SB202190. (d) SH-SY5Y cells were pretreated with 300 *μ*M antipain dihydrochloride (A.D.: cathepsins inhibitor) and/or 10 *μ*M SB202190 for 1 hour and subsequently incubated with 25 *μ*M TPEN for 12, 24, and 48 hours. Untreated cells were used as control and cells treated with 25 *μ*M TPEN were used as positive control of cell death in these cases. The Western blot images represent three independent experiments, and significant differences between treated and untreated cells were ^*∗*^ *p* < 0.05 and ^*∗∗*^ *p* < 0.001; and significant differences between treated and 25 *μ*M TPEN treated cells were ^#^ *p* < 0.05 and ^##^ *p* < 0.001.

**Figure 6:**
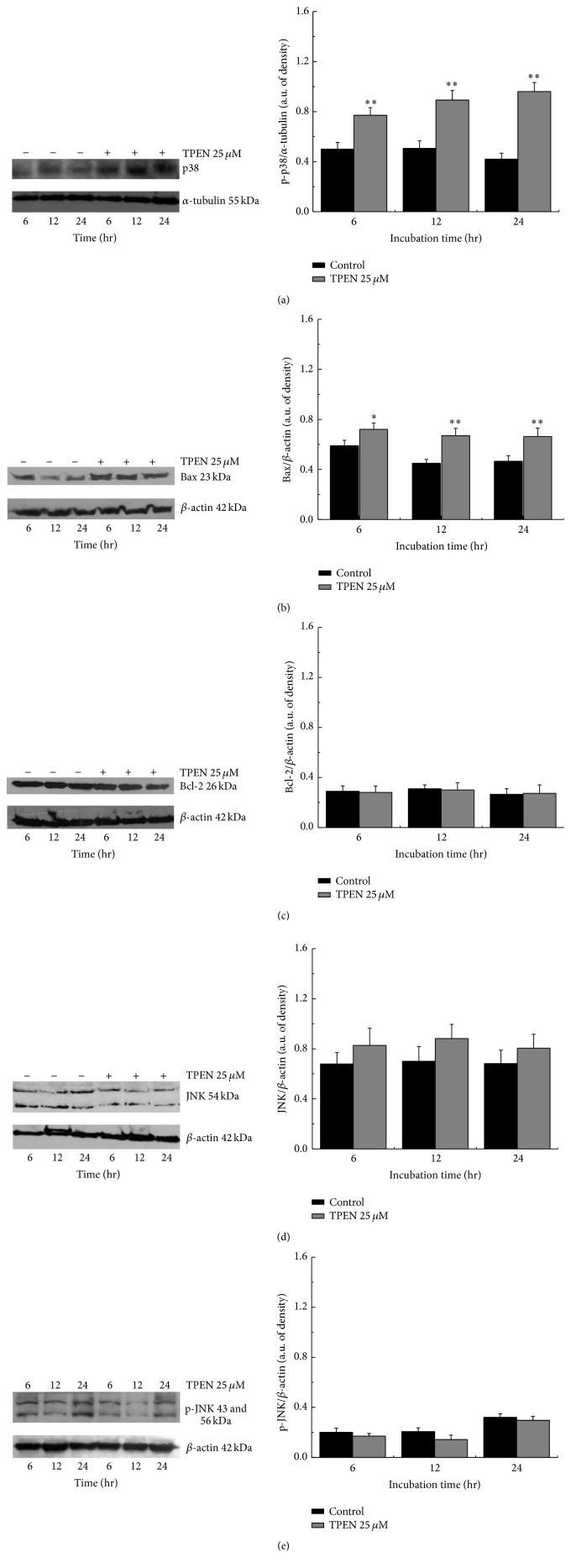
Influence of the TPEN chelator in the expression levels of apoptotic proteins. SH-SY5Y cells were treated with 25 *μ*M TPEN. At each time point, 100 *μ*g of total proteins from the total cell lysates was loaded onto each lane for the detection of p-p38 (a), Bax (b), Bcl-2 (c), JNK (d), and p-JNK (e). *β*-actin and *α*-tubulin were used as a loading control. The Western blot images represent three independent experiments, and significant differences between untreated and treated cells were ^*∗*^ *p* < 0.05 and ^*∗∗*^ *p* < 0.001, respectively.

Conversely, we analyzed the expression levels of another mitogen-activated protein kinase, p38 MAP kinase, and the results showed an increase compared with control experiments at all incubation times in the extracts obtained from cells (Figures 5(a) and 6(a)). We therefore used a p38 inhibitor to determine whether the activation of the cascade of apoptosis was first regulated by p38 MAP kinase. The compound SB202190 is a pyridinyl imidazole inhibitor that inhibits the activity of p38 MAP kinase through competition with ATP [46] and inhibits the phosphorylation of the protein [56]. When treating cells with an inhibitor of p38 MAP kinase, the highly selective and permeable membrane compound SB202190, it was observed that the compound completely inhibited the expression of both p38 MAP kinase and caspase 3; an increase in cell viability was also observed (Figure 5(d)), indicating the influence of p38 MAP kinase on the subsequent activation of caspase 3 (Figure 5(c)). Additionally, the expression levels of caspase 3 were analyzed, and the results showed that cells with zinc depletion also had an increased expression of this protein, particularly at 24 hours of incubation (Figure 5(b)).

We analyzed the expression levels of Bcl-2 protein by Western blot and observed no changes in Bcl-2 expression in the cell lysates (Figure 6(c)). However, the expression levels of Bax presented an increase following 12 hours of incubation (Figure 6(b)), because it is known that the c-Jun N-terminal kinase (JNK), one of the major mitogen-activated protein kinases (MAPKs), is also involved in cell death via either necrosis [53] or apoptosis [52]. The expression levels of this protein were also analyzed. The results obtained by Western blot showed that the expression levels of total JNK and phosphorylate JNK were not significantly affected when cells suffer zinc depletion with TPEN treatment (Figures 6(d) and 6(e)). It was already reported that JNK stimulates ROS formation, stimulating the protein ferritin to release iron and increase ferric ions in cytoplasm [45]. This increase consequently generates the rupture lysosomal cathepsins and releases the ferric ions to the environment, thus activating the genetically regulated necrosis [43]. However, our results showed that there is neither generation of ROS nor activation of JNK, suggesting that the observed lysosomal rupture is related only to the unbalance of intracellular zinc, which consequently contributes to a predominance of necrotic cell death pathway.

To conclude, our results showed that there is no generation of ROS (Figure 3), but there is a rupture of the lysosomal membrane (Figure 4). The experiments suggest that the rupture of lysosomes is related only to intracellular zinc imbalance. As TPEN induces the both events, activation of p38 MAPK and lysosomal rupture, we suggest that they occur independently of each other. We also observed that the activation of p38 MAP kinase only triggers the activation of the proapoptotic protein Bax. This may inactivate the antiapoptotic protein Bcl-2 and activate Bax protein interconnecting the apoptosis pathway with the lysosomal disturbance [57]. Therefore, because we observed a modest activation of Bax and no changes in the Bcl-2 in cells treated with zinc chelator TPEN, it is suggested that the depletion of intracellular zinc can influence the breakdown of lysosomes as a result of a release of cathepsins.

## Supplementary Material

Data from MTT experiments.
