# Supplementary material for: Zinc Chelation Mediates the Lysosomal Disruption without Intracellular ROS Generation
Source: Oxid Med Cell Longev. 2016 Mar 30;2016:6724585. doi: 10.1155/2016/6724585 (PMC4829717; doi:10.1155/2016/6724585)
Supplement: Supplementary file 1 — Data from MTT experiments. [file 6724585.f1.pdf]

Supporting Information

Figure 1A: Viability of human SH-SY5Y neuroblastoma cells with TPEN treatment.

1A: Viability by MTT method

<u>*Cells treated with TPEN and incubated for 12 hours.*</u>

	Control	5 µM	10 µM	25 µM	50 µM	100 µM
OD - 1	0.324	0.272	0.198	0.149	0.159	0.165
OD -2	0.392	0.317	0.295	0.211	0.184	0.132
OD - 3	0.405	0.332	0.308	0.198	0.166	0.207
OD - 4	0.426	0.317	0.267	0.257	0.258	0.203
OD - 5	0.441	0.318	0.321	0.281	0.282	0.161

Viability of each independent experiment= $\frac{OD.treated}{OD.control} x100$

	Control	5 µM	10 µM	25 µM	50 µM	100 µM
V.1	100	83.95062	61.11111	45.98765	49.07407	50.92593
V.2	100	80.86735	75.2551	53.82653	46.93878	33.67347
V.3	100	81.97531	76.04938	48.88889	40.98765	51.11111
V.4	100	74.41315	62.67606	60.32864	60.56338	47.65258
V.5	100	72.10884	72.78912	63.71882	63.94558	36.50794
Mean	100	78.66305	69.57615	54.55011	52.30189	43.97421
SD		5.118781	7.136956	7.472371	9.630958	8.286173
ANOVA Test		1.43148E-5	1.21256E-5	8.2134E-7	3.94307E-6	3.62466E-7

<u>*Cells treated with TPEN and incubated for 24 hours.*</u>

	Control	5 µM	10 µM	25 µM	50 µM	100 µM
OD - 1	0.451	0.271	0.277	0.071	0.189	0.201
OD -2	0.805	0.404	0.278	0.106	0.234	0.215
OD - 3	0.571	0.381	0.279	0.092	0.197	0.219
OD - 4	0.333	0.131	0.198	0.098	0.163	0.195
OD - 5	0.825	0.345	0.247	0.167	0.248	0.177

Viability of each independent experiment= $\frac{OD.treated}{OD.control} x100$

	Control	5 µM	10 µM	25 µM	50 µM	100 µM

V.1	100	60.08869	61.41907	15.74279	41.90687	44.56763
V.2	100	50.18634	34.53416	13.1677	29.06832	26.70807
V.3	100	66.72504	48.86165	16.11208	34.50088	38.35377
V.4	100	39.33934	59.45946	29.42943	48.94895	58.55856
V.5	100	41.81818	29.93939	20.24242	30.06061	21.45455
Mean	100	51.63152	46.84275	18.93889	36.89713	37.92851
SD	0	11.71352	14.25615	6.388722	8.427844	14.72368
ANOVA Test		$1.53x10^{-5}$	$3.24x10^{-5}$	$2.57x10^{-9}$	$1.64x10^{-7}$	$1.32x10^{-5}$

Cells treated with TPEN and incubated for 48 hours.

	Control	5 µM	10 µM	25 µM	50 µM	100 µM
OD - 1	0.649	0.742	0.312	0.201	0.15	0.119
OD -2	0.641	0.725	0.282	0.169	0.14	0.11
OD - 3	0.641	0.766	0.334	0.233	0.179	0.151
OD - 4	0.635	0.695	0.354	0.217	0.175	0.125
OD - 5	0.685	0.685	0.426	0.201	0.191	0.127

Viability of each independent experiment= $\frac{OD.treated}{OD.control} x100$

	Control	5 µM	10 µM	25 µM	50 µM	100 µM
V.1	100	114.3297	48.07396	30.97072	23.11248	18.3359
V.2	100	113.1045	43.99376	26.36505	21.84087	17.16069
V.3	100	119.5008	52.10608	36.34945	27.92512	23.55694
V.4	100	109.4488	55.74803	34.17323	27.55906	19.68504
V.5	100	100	62.18978	29.34307	27.88321	18.54015
Mean	100	111.2768	52.42232	31.44031	25.66415	19.45574
SD		7.259521	7.009146	3.93528	2.947683	2.461304
ANOVA Test		0.0084	$3.52x10^{-7}$	$2.07x10^{-10}$	$1.09x10^{-11}$	$1.36x10^{-12}$
